# Efficacy of Intravenous Colistin Monotherapy Versus Colistin Combined With Meropenem in Patients With Multidrug-Resistant Infections: A Retrospective Observational Study

**DOI:** 10.7759/cureus.47342

**Published:** 2023-10-19

**Authors:** Mehwish Baig, Sana Rahim, Rashid Naseem Khan, Dilroz Dilara Memon, Zaid A Ansari, Muhammad Athar Khan

**Affiliations:** 1 Intensive Care Unit, Liaquat College of Medicine and Dentistry, Darul Sehat Hospital, Karachi, PAK; 2 Internal Medicine, Liaquat College of Medicine and Dentistry, Darul Sehat Hospital, Karachi, PAK; 3 Medical School, Liaquat College of Medicine and Dentistry, Karachi, PAK; 4 Community Medicine, Liaquat College of Medicine and Dentistry, Karachi, PAK

**Keywords:** retrospective, intensive care unit (icu), mdr infections, colistin-meropenem, monotherapy, colistin

## Abstract

Background

Intensive care units frequently contend with infections caused by highly drug-resistant organisms, particularly Acinetobacter baumannii, Pseudomonas aeruginosa, Klebsiella pneumoniae, and Enterobacterales (CRE), which often lead to high mortality rates. Colistin (colomycin) is employed to treat infections, notably extremely drug-resistant (XDR) bacteria. Antibiotic combination treatment is a frequently used tactic in this endeavour. However, the widespread use of antibiotics in synergy could result in the emergence of resistance and a rise in side effects, such as those linked to Clostridium difficile infection. The aim of the study was to assess and contrast the clinical results of intravenous colistin monotherapy with the combination of colistin and meropenem in patients experiencing MDR bacteremia resulting from Acinetobacter Baumannii, Pseudomonas aeruginosa, Klebsiella pneumoniae, and Enterobacterales (CRE).

Methods

In this retrospective observational study, an analysis spanning two years, from June 2021 to June 2023, was conducted at a teaching hospital located in Karachi, Pakistan. The research involved the retrospective examination of medical records from 132 patients who had been diagnosed with MDR bacteremia. Patients were divided into two categories based on their treatment regimen, either intravenous colistin monotherapy or intravenous colistin combined with meropenem. Among the 132 patients included in the analysis, 66 underwent colistin monotherapy, while the other 66 received a combination of colistin and meropenem. The primary focus of evaluation in this study centered on the 14-day all-cause mortality, while secondary outcomes encompassed clinical success and microbiologic cure.

Results

The mean age of patients in both groups was comparable, and there were no noteworthy gender differences. Additionally, the distribution of infection types and the isolated pathogens showed no substantial distinctions between the two groups. The study revealed no statistically significant disparities in 14-day mortality, improvement in Sequential Organ Failure Assessment (SOFA) score, or the proportion of patients who were cured and survived between the two treatment groups.

Conclusion

The findings from this study lead to the conclusion that there exists no significant disparity in the efficacy of colistin monotherapy compared to the combination of colistin with meropenem in the treatment of MDR bacteremia stemming from Acinetobacter Baumannii, Pseudomonas aeruginosa, Klebsiella pneumoniae, and Enterobacterales (CRE). The results provide a basis for future research and underscore the significance of ongoing endeavors to refine antibiotic treatment strategies in response to the worldwide issue of antibiotic resistance.

## Introduction

Intensive care units frequently confront infections caused by highly drug-resistant organisms, particularly Acinetobacter baumannii [1], Pseudomonas aeruginosa, Klebsiella pneumoniae, and Enterobacterales (CRE), which often result in elevated mortality rates. To effectively address and manage these critical threats to human health, there is a pressing need for the development of new drugs [2-4]. Colistin, also known as colomycin, emerges as a powerful polypeptide antibiotic that acts externally on the lipopolysaccharide of gram-negative bacteria. It is primarily utilized to combat infections, notably those classified as extremely drug-resistant (XDR), caused by bacteria such as Acinetobacter baumannii, Pseudomonas aeruginosa, Klebsiella pneumoniae, and meropenem-resistant strains of Enterobacter. One notable characteristic of colistin is its possession of both hydrophilic and lipophilic properties.

Questions regarding the effectiveness of colistin monotherapy have arisen, prompting efforts to enhance treatment success rates. A frequently employed strategy in this pursuit is antibiotic combination therapy. This approach capitalizes on colistin's ability to enhance bacterial outer membrane permeability, facilitating the entry of larger hydrophobic compounds [5]. Despite certain in vitro findings, combination therapy is also believed to curtail resistance development and sustain efficacy against Acinetobacter infections [6]. However, the efficacy of colistin-based combination therapy in treating multidrug-resistant (MDR) Acinetobacter infections has demonstrated inconsistent results in clinical trials. Moreover, the widespread use of antibiotics in synergy could potentially lead to resistance emergence and an increase in side effects, including those associated with Clostridium difficile infection.

The efficacy of colistin monotherapy initially raised questions, prompting researchers to investigate combination therapy with carbapenem or sulbactam to achieve a synergistic effect. Several in vitro studies explored the potential of sulbactam, fosfomycin, tigecycline, and meropenem in conjunction with colistin [7-9]. However, the success of combination therapy based on colistin has yielded conflicting results in clinical trials. In an effort to address this divisive topic, two meta-analyses were conducted. The first, conducted by Zusman et al. in 2017 [10], and the second, by Vardakaset et al. in 2018 [11], aimed to provide clarity. It is worth noting that among the papers included in these meta-analyses, only three were randomized controlled trials (RCTs) [10-13], with the majority of the studies being retrospective observational studies. Consequently, the conclusions drawn in these meta-analyses [10,11] were based on evidence of relatively poor quality.

Despite numerous studies on the topic, the existing body of evidence supporting the effectiveness of colistin, whether administered alone or in combination, for treating critically ill patients with multidrug-resistant (MDR) infections remains limited [14]. The primary objective of this study was to assess and compare the clinical outcomes of two treatment modalities for patients with MDR infections caused by bacteria such as Acinetobacter baumannii, Pseudomonas aeruginosa, Klebsiella pneumoniae, and Enterobacterales (CRE): intravenous colistin monotherapy and intravenous colistin combined with meropenem.

## Materials and methods

This study conducted a retrospective analysis of panculture reports, including samples from urine, blood, and trachea, collected from Darul Sehat Hospital, a teaching hospital in Karachi, which has approximately 250 beds. Our evaluation focused on 132 cases of patients diagnosed with multidrug-resistant (MDR) bacteremia occurring between June 2021 and June 2023. Patients were divided into two groups: one group received intravenous colistin monotherapy, while the other group received a combination of intravenous colistin and meropenem. To eliminate potential bias stemming from recurrent episodes, we included only the initial occurrence of MDR bacteremia in patients experiencing multiple instances in our analysis.

This study considered individuals aged 18 years or older who had been diagnosed with MDR bacterial infections and had received either colistin monotherapy or a combination therapy involving meropenem as their primary treatment. Only patients admitted to the intensive care unit (ICU) were eligible for inclusion. The exclusion criteria were patients who had received different primary treatments, such as other antibiotics or combination therapies that did not include colistin and meropenem, as well as those who had been prescribed colistin or meropenem for preventive or non-infectious reasons. Furthermore, the analysis excluded pediatric patients, pregnant individuals, cases involving pathogens not under study, and patients who had passed away within 48 hours of starting treatment.

This study required a total sample size of 132 individuals. To ascertain the specified difference (at the required confidence level and power), a minimum sample size of 66 participants was established for each group. The calculation of the required sample size was based on assumptions derived from reported mortality rates by Abdelsalam et al., indicating 16.7% in the colistin group and 43.3% in the colistin with meropenem group, with 80% statistical power and a 95% confidence level [15]. The sample size computation was performed using the EpiTools online sample size calculator (http://epitools.ausvet.com.au).

Clinical data pertaining to patients with MDR bacteremia were retrieved from medical records. This data included initial baseline characteristics such as age, gender, vital signs, underlying medical conditions, and the primary site of infection. Additionally, clinical data regarding antimicrobial treatment and mortality outcomes were collected throughout the entire clinical course. Blood, tracheal, and urine specimens provided by patients underwent immediate microbiological testing. These specimens were placed in a BACT-ALERT 3D instrument for incubation [16], and bacterial organisms isolated from cultures showing positive results underwent traditional overnight identification and sensitivity panels using Blood Agar and MacConkey Agar through a four-way streaking method. The antibiotic classes tested included ampicillin/sulbactam, aztreonam, cephalosporins (including three third-generation and one fourth-generation), aminoglycosides (gentamicin and tobramycin), quinolones (ciprofloxacin and levofloxacin), carbapenems (imipenem and meropenem), tetracycline, and trimethoprim/sulfamethoxazole. Isolates were assessed accordingly.

Patients received colistin methanesulfonate alone or in combination with meropenem as part of their treatment regimen. This treatment commenced with a 9-million unit (MIU) colistin methanesulfonate loading dose, followed by 4.5-MIU maintenance doses administered every 12 hours, adjusted based on renal function [17]. Additionally, a 1 g infusion of meropenem [18] was given every eight hours following the established protocol [19]. Doses for both drugs were adjusted based on renal function. While patients could receive additional antibiotics targeting gram-positive or anaerobic co-infections, the use of antibiotics specifically targeting gram-negative bacteria through systemic or inhalation routes was not permitted. Patient enrollment in the trial depended on the identification and susceptibility testing of the initial isolates conducted in the local laboratory.

The primary outcome under examination in this study was the 14-day all-cause mortality, with secondary outcomes including clinical success evaluated at 14 days from the onset of infection and microbiologic cure. Patients who did not fulfill all the specified success criteria were categorized as experiencing clinical failure. Success was defined as a combination of factors, including the patient being alive, maintaining stable hemodynamics (systolic blood pressure >90 mm Hg without requiring vasopressor support), and demonstrating improvement or stability in the Sequential Organ Failure Assessment (SOFA) score. For patients initially having a SOFA score ≥3, an improvement of at least 30% in the score was necessary, while for those with a baseline SOFA score <3, the score needed to remain unchanged or decrease [6]. Furthermore, in cases of pneumonia, achieving a stable or improved ratio of partial pressure of arterial oxygen to fraction of expired oxygen was considered. Additionally, for patients with bacteremia, a successful outcome required a microbiological cure, defined as the absence of growth of the initial isolate in the blood on Day 14 or later.

Continuous variables were expressed as mean ± standard deviation, while categorical variables were represented as frequencies and percentages. To compare the 14-day mortality between both groups, Pearson's chi-squared test was employed. All significance testing followed a two-tailed approach, and p-values less than 0.05 were regarded as statistically significant. The statistical analyses were conducted using SPSS for Windows v. 23.0 (IBM Corp., Armonk, NY). This study adhered to established ethical standards and regulations, obtaining all necessary approvals from the institutional review board (IRB) of Liaquat College of Medicine and Dentistry, (IRB/M-000062/23). To safeguard confidentiality, patient data utilized in this retrospective study were anonymized, and the need for informed consent was waived due to the retrospective nature of the study.

## Results

The demographics and clinical traits of two groups - one treated with colistin alone and the other with colistin and meropenem are summarised in Table 1. The average patient age in the colistin monotherapy group was 64 years with a standard deviation of 6, while it was 63 years with a standard deviation of 5 in the colistin + meropenem combination group (p = 0.300). In total, 32 patients (48.5%) were female and 34 (51.5%) were male in the colistin monotherapy group, and 32 patients (48.5%) and 34 patients (51.5%) in the colistin + meropenem combination group were male and female, respectively (p = 0.728).

**Table 1:** Comparison of demographics and clinical characteristics of patients treated with either colistin monotherapy or colistin + meropenem combination (n=132) SD, standard deviation; SOFA, Sequential Organ Failure Assessment

	Colistin (n=66 )	Colistin plus Meropenem (n=66 )	p-value
Demographic data Age, years (mean + sd)	64 + 6	63 + 5	0.300
Gender, n (%) Male	34(51.5)	32(48.5)	0.728
Type of Infection Blood Stream Infection (Yes)	13(19.7)	12(18.2)	0.824
Pneumonia (Yes)	09(13.6)	13(19.7)	0.350
UTI (Yes)	25(37.9)	21(31.8)	0.465
Pathogen Isolated Acinetobacter Baumannii (Yes)	05(7.6)	08(12.1)	0.380
Klebsiella (Yes)	09(13.6)	07(10.6)	0.594
Pseudomonas (Yes)	02(3)	05(7.6)	0.244
Escherichia Coli (Yes)	16(24.2)	11(16.7)	0.281

Examining infection types, in the colistin monotherapy group, 13 patients (19.7%) had bloodstream infections, while 53 (80.3%) did not. In the colistin + meropenem combination group, 12 patients (18.2%) had bloodstream infections, while 54 (81.8%) did not (p = 0.824). Furthermore, in the colistin monotherapy group, nine patients (13.6%) had pneumonia, while 57 (86.4%) did not. In the colistin + meropenem combination group, 13 patients (19.7%) had pneumonia, while 53 (80.3%) did not (p = 0.350). Regarding urinary tract infections (UTIs), among the patients in the colistin monotherapy group, 25 (37.9%) had UTIs, while 41 (62.1%) did not. In the colistin plus meropenem combination group, 21 (31.8%) had UTIs, while 45 (68.2%) did not (p = 0.465).

In terms of isolated pathogens, five patients (7.6%) in the colistin monotherapy group had Acinetobacter baumannii, nine (13.6%) had Klebsiella, two (3%) had Pseudomonas, and 16 (24.2%) had Escherichia coli. In the colistin + meropenem combination group, eight (12.1%) had Acinetobacter baumannii, seven (10.6%) had Klebsiella, five (7.6%) had Pseudomonas, and 11 (16.7%) had Escherichia coli. Notably, there were no statistically significant differences in the distribution of these pathogens between the two groups.

Table 2 presents a comparison of mortality and clinical morbidity outcomes in patients treated with colistin monotherapy versus colistin + meropenem combination therapy. Within the colistin monotherapy group, 34 patients (51.5%) demonstrated an improvement in the Sequential Organ Failure Assessment (SOFA) score, while in the colistin + meropenem combination group, 26 patients (39.4%) exhibited improvement. Nevertheless, there was no statistically significant difference observed in the improvement of the SOFA score between the two treatment groups (p = 0.162).

**Table 2:** Comparison of mortality and clinical morbidity outcomes in patients treated with colistin monotherapy versus colistin + meropenem combination therapy (n=132). Statistical analysis was carried out using chi-square test

Clinical Outcome n (%)	Colistin (n=66 )	Colistin plus Meropenem (n=66 )	p-value
Improvement in SOFA Score (Yes)	34(51.5)	26(39.4)	0.162
14^th^ Day Mortality (Yes)	32(48.5)	40(60.6)	0.162
Cured & Survived (Yes)	34(51.5)	26(39.4)	0.162

**Figure 1:**
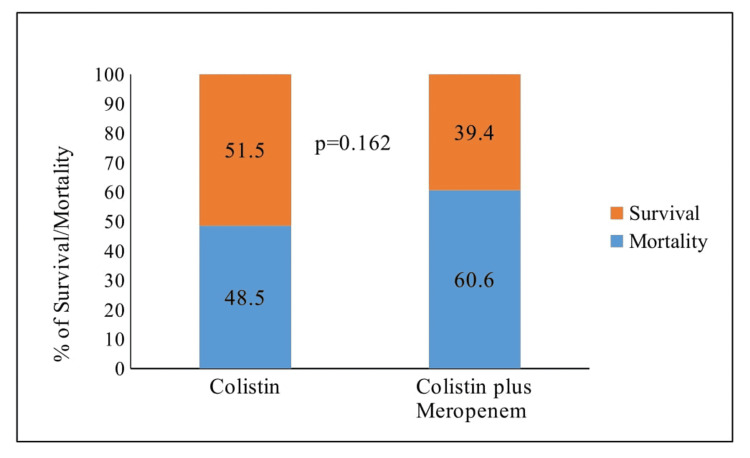
Survival/mortality in the colistin monotherapy group versus the colistin + meropenem combination therapy group

Furthermore, in the colistin monotherapy group, 32 patients (48.5%) experienced mortality by the 14th day, while in the colistin + meropenem combination group, 40 patients (60.6%) also experienced mortality (p = 0.162). Additionally, within the colistin monotherapy group, 34 patients (51.5%) were cured and survived, whereas in the colistin + meropenem combination group, 26 patients (39.4%) were cured and survived. Once again, no statistically significant difference was noted in this aspect (p = 0.162) (Figure 1).

## Discussion

The objective of this retrospective study was to assess and compare the clinical outcomes of patients with multidrug-resistant (MDR) infections who received either colistin monotherapy or a combination therapy of colistin and meropenem. The mean age of patients in both groups was quite similar, with an average age of 64±6 years for the colistin monotherapy group and 63±5 years for the colistin + meropenem combination group (p = 0.300). The gender distribution in both groups was nearly equal group (p = 0.728). In the colistin monotherapy group, 19.7% of patients had bloodstream infections, 13.6% had pneumonia, and 37.9% had urinary tract infections (UTIs). Conversely, in the colistin + meropenem combination group, 18.2% had bloodstream infections, 19.7% had pneumonia, and 31.8% had UTIs (p > 0.05). Acinetobacter baumannii, Klebsiella, Pseudomonas, and Escherichia coli were the most frequently isolated pathogens in the colistin monotherapy group, accounting for 7.6%, 13.6%, 3%, and 24.2% of cases, respectively. In the colistin + Meropenem combination group, 8 (12.1%) had Acinetobacter baumannii, 7 (10.6%) had Klebsiella, 5 (7.6%) had pseudomonas, and 11 (16.7%) had Escherichia coli (p > 0.05). Furthermore, age, gender, illness types, and isolated pathogens did not differ significantly between the two groups.

The clinical outcomes observed in both treatment groups showed no statistically significant disparities, as evidenced by p-values of 0.162 for improvement in the Sequential Organ Failure Assessment (SOFA) score, 14th-day mortality, and the percentage of patients who were cured and survived. These outcomes suggest that, within our study population, colistin monotherapy and colistin + meropenem combination therapy exhibited similar effectiveness in treating MDR infections caused by Acinetobacter baumannii, Pseudomonas aeruginosa, Klebsiella pneumoniae, and Enterobacterales (CRE). However, it's important to note that while statistically significant differences weren't found, the observed percentages of clinical outcomes imply some variations between the two treatment groups. Consequently, further research [20] with larger sample sizes and well-designed studies may be required to arrive at more definitive conclusions regarding the comparative effectiveness of these treatment options.

Our findings align with prior research, which has also reported conflicting results concerning the superiority of combination therapy over monotherapy in treating MDR infections. While certain in vitro studies [7-9] have suggested that combination therapy might be more effective due to the increased permeability effect of colistin on bacterial outer membranes, clinical trials have yielded inconclusive results. Our study, encompassing a diverse patient population over a two-year period, supports the notion that both treatment approaches may be viable options for managing MDR infections. The choice between them may depend on various factors, including patient-specific characteristics, drug availability, and local antibiotic resistance patterns.

In the context of the escalating threat of antibiotic resistance, colistin, an older antibiotic, has emerged as a critical last-resort option for treating severe MDR infections. The observed efficacy of colistin monotherapy in our study underscores its relevance as a valuable treatment option. However, the results also underscore the importance of prudent antibiotic usage and the need to avoid widespread administration of antibiotics to prevent the emergence of further resistance [21] and adverse effects such as Clostridium difficile-associated disease [22].

It is important to acknowledge certain limitations inherent in this research. Firstly, the study's retrospective nature may introduce inherent biases and limitations associated with data availability and completeness. This study had a relatively small sample size and the single-center design might limit the generalizability of the findings to broader patient populations. Finally, the study's follow-up period of 14 days may not capture longer-term outcomes or variations in patient response beyond this timeframe. Given these limitations, further research, including larger-scale multicenter studies and randomized controlled trials, is warranted to provide more comprehensive insights into the comparative effectiveness of these treatment modalities for MDR infections.

## Conclusions

In conclusion, our study offers valuable insights into the management of MDR infections by comparing colistin monotherapy with colistin + meropenem combination therapy. The absence of statistically significant differences in clinical outcomes suggests that both approaches may constitute viable options. However, the decision should be individualized, taking into account patient-specific factors and local resistance patterns. These findings establish a foundation for further research and underscore the significance of ongoing efforts to optimize antibiotic treatment strategies in response to the global challenge of antibiotic resistance.
